# Robust Multimodal Emotion Recognition from Conversation with Transformer-Based Crossmodality Fusion

**DOI:** 10.3390/s21144913

**Published:** 2021-07-19

**Authors:** Baijun Xie, Mariia Sidulova, Chung Hyuk Park

**Affiliations:** Department of Biomedical Engineering, School of Engineering and Applied Science, George Washington University, Washington, DC 20052, USA; bdxie@gwu.edu (B.X.); sidul001@gwu.edu (M.S.)

**Keywords:** multimodal emotion recognition, multimodal fusion, crossmodal transformer, attention mechanism

## Abstract

Decades of scientific research have been conducted on developing and evaluating methods for automated emotion recognition. With exponentially growing technology, there is a wide range of emerging applications that require emotional state recognition of the user. This paper investigates a robust approach for multimodal emotion recognition during a conversation. Three separate models for audio, video and text modalities are structured and fine-tuned on the MELD. In this paper, a transformer-based crossmodality fusion with the EmbraceNet architecture is employed to estimate the emotion. The proposed multimodal network architecture can achieve up to 65% accuracy, which significantly surpasses any of the unimodal models. We provide multiple evaluation techniques applied to our work to show that our model is robust and can even outperform the state-of-the-art models on the MELD.

## 1. Introduction

In recent years, there has been a growing number of studies that have attempted to recognize human emotion from either speech [1,2,3], text [4,5], or facial expressions [6,7]. In reality, emotional communication is a temporal and multimodal process; typical human conversations consist of a variety of cues and expressions that are rarely static. Thus, multiple studies have highlighted the importance of multisensory integration when processing human emotions [8,9,10,11].

Emotion recognition has extensive application prospects, including but not limited to Human–Robot Interaction (HRI), Socially Assistive Robotics (SAR), Human–Computer Interaction (HCI), and medicine. Discussion regarding the effectiveness of multimodal HRI has dominated the research in recent years. For example, in the paper by Stiefelhagen et al. [12], the researchers discussed a novel multimodal HRI system, which included speech recognition, multimodal dialogue processing, visual detection, tracking, and identification of users, which combined both head pose estimation and pointing gesture recognition. The study with human participants concluded that the incorporation of all of these modalities increased participants’ engagement and made the HRI scenario more natural. For Socially Assistive Robots (SARs) to effectively communicate with human beings, robotic systems should have the ability to interpret human affective cues and to react appropriately by exhibiting their own emotional response. In the work by Hong et al. [13], the researchers presented a multimodal emotional HRI architecture to assist in natural, engaging, bidirectional emotional communication between humans and a robot. Both body language and vocal intonation were measured to recognize the user’s affective state. The results of the experiment with human participants proved that bidirectional emotion recognition instigated more positive valence and less negative arousal during the interaction. Kim et al. [14] developed an audio-based emotion recognition system that can estimate the expression levels for valence, arousal, and dominance. The extracted features from the speech data were used to train the automatic emotion classifier. This classifier offers emotional communications in a natural manner during human–robot interaction experiences for children with Autism Spectrum Disorder (ASD). More recently, the study of [15] also applied deep learning methods to recognize emotion from the music. Several deep learning networks were employed to train the extracted spectral features from the music and predict the levels of arousal and valence.

Emotion recognition has also been heavily studied in the context of Human–Computer Interaction (HCI). Creating human–computer interaction that would be as natural and efficient as human–human interaction requires not only recognizing the emotion of the user, but also expressing emotions. An example of such research is the work of Maat and Pantic [16], where the authors proposed a system that is capable of learning and analyzing the user’s context-dependent behavior and adapting the interaction to support the user. Another example of an HCI system with emotion recognition is the Intelligent Tutoring System developed by Kapoor et al. [17], which incorporated multisensory data to assist in detecting frustration to predict when the user needs help. Emotion-oriented HCIs aim to not only automatically recognize emotional states, but also mimic or synthesize emotions in speech or facial expressions. As an example, previous studies focused on generating voices that would have relevant emotional sentiment [18,19]. Learning emotions from the speech provides a way of generating convincing emotional speech [20].

Medical specialists can also benefit from using emotion recognition systems as a diagnostic tool for a wide range of medical symptoms. For example, in the study by France et al. [21], the researchers compared the acoustic trends in the speech of healthy, depressed, and suicidal individuals. Another medical example is clinical studies of schizophrenia and flat affect, which is an indicative symptom of the disease and is characterized as diminished emotional expression [22,23].

In general, user affect is detected using a unique combination of body language and vocal intonation, and multimodal classification is performed using computational models, e.g., a Bayesian network [24,25]. When applied in the robotics domain, human emotion recognition in the HRI system can especially make the interaction more natural, understandable, and intuitive [26]. In the study by Barros et al. [27], a cross-channel Convolutional Neural Network (CNN) structure was proposed to investigate how emotions are expressed by a robotic system and changed the perception of human users. The network was able to predict human emotions using features based on facial expressions and body motions. The emotion recognition system was tested in a real HRI scenario with the iCub robot. The robot was able to detect three different emotional states and gave feedback by changing its mouth and eyebrow LEDs. In another study by Javed et al. [28], the robotic system was trained to recognize emotion states from both typically developing children and children with ASD. With the goal of measuring the overall engagement of the child during the HRI session, emotion recognition was used as one of the measured features. Therefore, it is believed that advancing emotion recognition from human beings can significantly improve the role of empathy in HRI scenarios as well.

In this study, a robust transformer-based multimodal fusion network for emotion recognition is presented. The embedding vectors from each individual modality were extracted from domain-specific models and fused via our proposed crossmodality transformer. In addition to considering the joint representation across different modalities, we introduce a robust multimodal fusion network to combine all the representation vectors from each modality. The results reached state-of-the-art performance on the evaluated dataset.

## 2. Related Studies

### 2.1. Multimodal Fusion for Emotion Recognition

The primary fusion strategies from previous studies for multimodal emotion recognition can be classified into feature-level (early) fusion, decision-level (late) fusion, and model-level fusion [29]. Conventionally, feature-level fusion concatenates the features from different modalities to obtain a joint representation, and the concatenated features are fed into a single classifier for emotion recognition. Schuller et al. [30] presented baseline models, which concatenated the audio and visual features into a single feature vector and used support vector regression to predict the continuous affective values. A recent study [31] also investigated using a Long Short-Term Memory Recurrent Neural Network (LSTM-RNN) to train different modalities of features and to combine different feature vectors via concatenation. However, feature-level fusion may suffer from the problem of data sparseness [29], so the performance of combining different modalities via a simple concatenation is limited.

Unlike feature-level fusion, decision-level fusion employs and trains separate classifiers for each modality and combines the outputs from each classifier to obtain the final prediction. Liu et al. [32] applied different kernel-based classifiers for different modalities and boosted the fusion results at the decision level. However, decision-level fusion does not consider the mutual relations between different modalities, which results in losing correlated information among different modalities.

Researchers have also taken advantage of deep learning models for model-level fusion, which also consider the interrelations between different modalities. Chen et al. [33] achieved model-level fusion by concatenating the high-level features from different modalities. More recently, Choi et al. [34] proposed a novel multimodal fusion architecture based on a neural network for classification tasks. The model architecture ensured both the effectiveness of the crossmodal relationship and the robustness of fusing different modalities.

### 2.2. Transformer Method

Multimodal Emotion Recognition (MMER) with fusion by the transformer has drawn much attention recently. The transformer is a network architecture that purely depends on the attention mechanism without any recurrent structure [35]. The latest studies focused on using attention mechanisms to fuse different modalities of features for MMER [36,37,38,39]. Ho et al. [36] proposed a multimodal approach based on a multilevel multi-head fusion attention mechanism and RNN to combine audio and text modalities for emotion estimation. The paper also stated that the method of attention mechanism fusion for multiple modalities improved the emotion recognition performance compared to the unimodal approach. Another study by Huang et al. also compared decision-level and feature-level fusions by using the attention mechanism [37]. This study utilized the transformer to fuse audio and visual modalities at the multi-head attention level. The experiments showed that the proposed method had better performance via feature-level fusion.

The previous study investigated the use of the crossmodal transformer to reinforce a target modality by introducing the features from another modality, which also learns the attention across these two modalities’ features [40]. One recent study [39] proposed a multimodal learning framework based on the crossmodal transformer target for conversational emotion recognition, combining word-level features and segment-level acoustic features as the inputs. The results demonstrated the effectiveness of the proposed transformer fusion method. Another recent study [38] combined three different modalities, text, audio, and vision, with features extracted from the pre-trained self-supervised learning model. This study designed a transformer-based multimodal fusion mechanism that also considered the intermodality connections and achieved state-of-the-art results for the task of MMER.

Even though the effectiveness of combining two different modalities by using the attention mechanism has been widely studied, the challenge emerges when the need arises to combine three or more modalities due to the structure of multi-head attention. For this reason, most previous studies on MMER based on the attention mechanism proposed and tested the network architecture for only two modalities [36,37,39]. The study [38] deployed models for combining three modalities with transformer-based fusion, but a simple feature concatenation was added at the end to combine different modalities’ features. However, in practice, concatenating different high-dimensional features may result in data sparseness and degrade the performance [29].

## 3. Dataset

**Figure 1:**
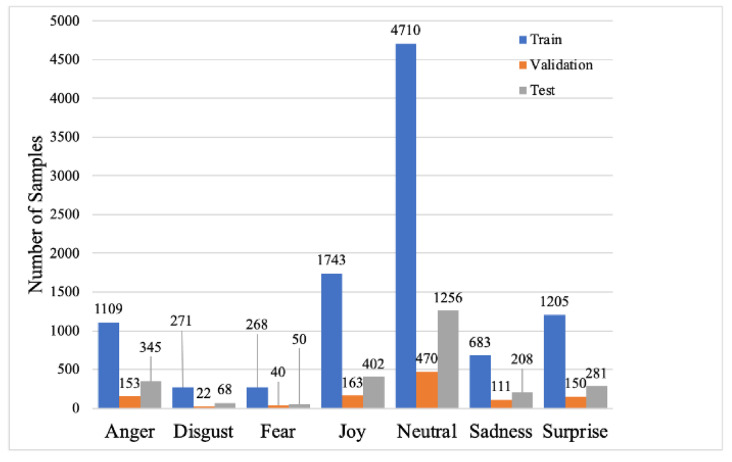
Number of samples per one emotion label for the training, validation, and test dataset.

The Multimodal Emotion Lines Dataset (MELD) [41] is an extended version of the EmotionLines dataset [42]. The MELD includes a total of 13,708 samples segmented out from the TV series *Friends*, with the samples grouped as 9989 for training, 1109 for validation, and 2610 for testing. Each segmented sample has the following data attributes, which were used in this study: video clip, utterance, text from the utterance, and emotion labels. There are seven emotion labels available for the dataset: *Anger*, *Disgust*, *Sadness*, *Joy*, *Neutral*, *Surprise*, and *Fear*. These are typically considered to be the basic emotions, and other emotions are seen either as combinations of these basic emotions as studied by Bower [43]. Label distributions in the training, validation and test datasets can be seen in Figure 1. There is an inherent imbalance in the MELD: *Neutral* samples dominate in each of the datasets. Utterance audio clips vary in time length, but are averaged to around 5 s-long recordings. On average, there are around seven words per one utterance. The average duration of an utterance is 3.95 s, and the average number of utterances per dialogue is 9.6. The number of emotions per dialogue is 3.3 on average.

## 4. Methods

In this study, three different deep learning models are introduced for feature extraction from three different modalities. The crossmodality fusion transformer approach and EmbraceNet for fusion are discussed for multimodal classification.

### 4.1. Feature Extraction

**Figure 2:**
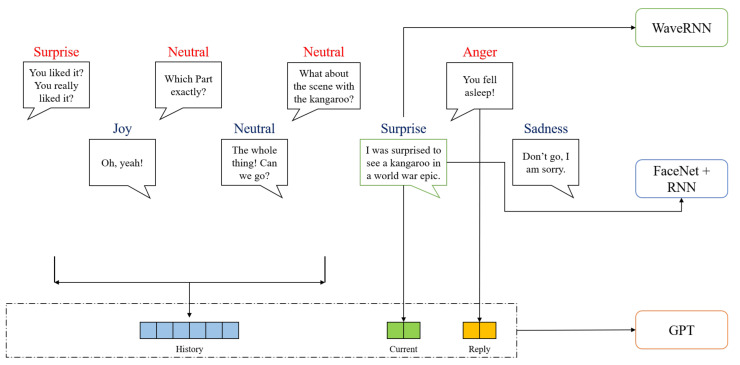
The crossmodality attention fusion transformer.

Three different deep neural networks were used for feature extraction: Generative Pre-trained Transformer (GPT), WaveRNN, and FaceNet+RNN. Transfer learning is a training scheme that provides help for training small dataset [44]. Transfer learning proposes first pre-training the model on a large-scale dataset, then for another smaller target dataset, the pre-trained model can be fine-tuned and achieve the desired performance efficiently. Therefore, the transfer learning paradigm was adopted in this study. In the stage of feature extraction, we first fine-tuned the domain-specific models for the emotion classification task with a single modality. Then, we extracted the features before the fully connected layer from the fine-tuned models, which were prepared for the fusion and training in the multimodal fusion stage. Figure 2 shows a dialogue sample from the conversation of two actors, and different modalities of features from the dialogue are fed into the corresponding models. The WaveRNN accepts the audio features from the sentence’s audio clip, and the sequential face images from the video clip are fed into the FaceNet+RNN model. For the text modality, the input consists of the dialogue of the history, current sentence, and reply.

#### 4.1.1. Model for Text Modality

We used the GPT [45] as our language model for extracting the features from the text modality. GPT is a multilayer transformer-based model, which was pre-trained on the BookCorpus dataset [46] and fine-tuned on the MELD dialogue dataset. As shown in Figure 2, the transfer learning scheme proposed by Wolf et al. [47] was adapted. Interested readers can refer to their repository (https://github.com/huggingface/transfer-learning-conv-ai, accessed on 18 July 2021) for more details. To estimate the emotional states of the current sentence, we combined the history of the dialogues, the current sentence, and the reply sentence to generate sentence embeddings. Then, the position embeddings and segment embeddings were summed up to build a single input sequence for the transformer model to obtain the output sequence tokens.

The optimization of the model was performed by multi-task learning. The fine-tuning was performed by introducing a combination of different loss functions: (1) next-sentence prediction loss, (2) language modeling loss, and (3) emotion classification loss. For the next-sentence prediction loss, L(s), a special token [CLS] is added at the end of the input sequence. The optimization goal was to train a classifier to discriminate the gold reply (ground-truth sentence) from the randomly sampled distractor (the sentence other than the ground-truth). The corresponding last hidden state for token [CLS] is passed to the linear classifier, and the cross-entropy loss is used to optimize the classifier. For the language modeling loss, L(l), the final hidden states of the outputs are extracted to predict the next reply tokens, and the cross-entropy loss is used to optimize the language model. For the emotion classification loss, L(e), a multilayer classifier is introduced for classifying seven emotion labels from the MELD. During training, the last hidden state from the final hidden layer is fed into the classifier to predict the emotions, and the cross-entropy loss is computed to optimize the emotion model. Finally, the total loss function for optimization is the sum of these losses:

(1) L(total)=L(s)+L(l)+L(e).

#### 4.1.2. Model for Audio Modality

The WaveRNN [48] model was used as an audio model to extract features from original audio clips. The original waveform of the audio and generated spectrogram of the signal are both inputs in the model. The pre-trained model used in this study was trained on LJSpeech dataset [49]. During the preprocessing, waveforms were sampled at a 16,000 Hz sample rate, and spectrograms were generated. Moreover, zero-padding for the batch inputs was applied on-the-fly during training. The input channels of the waveform and spectrogram have to be 1, so we averaged the values from both channels of the audio inputs. All the preprocessing was implemented via the torchaudio library (https://pytorch.org/audio/stable/index.html, accessed on 18 July 2021). As shown in Figure 2, the acoustic features from the time interval of one person’s speech were extracted as the inputs for the model.

**Figure 3:**
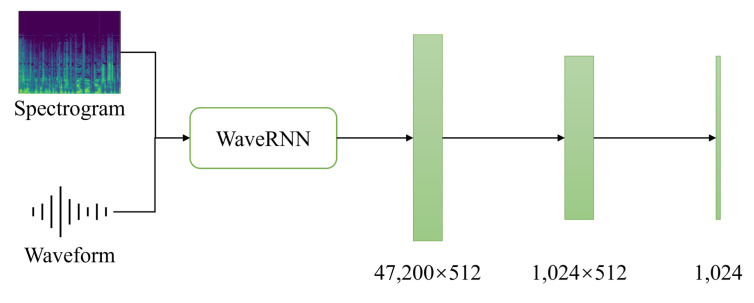
The classifier added to WaveRNN.

The input shapes for the WaveRNN can be defined as follows:

[batch_size,n_channel=1,feature_size=(n_time−kernel_size+1)×hop_length]

for the input waveform, and:

[batch_size,n_channel=1,n_freq,n_time]

for the input spectrogram, where hop_length=200, kernel_size=5, and n_time=240 in this study. As can be seen in Figure 3, the feature vector with the shape of [(n_time−kernel_size+1)×hop_length=47,200,512] before the fully connected layer of the WaveRNN model is extracted, where 512 is the default feature size of the WaveRNN output. The classifier consists of two consecutive linear layers at the end, giving us the predictions of the emotional states.

#### 4.1.3. Model for Face Modality

Deep CNN has been widely studied for facial emotion recognition from videos by extracting the sequence of face embeddings [50,51,52]. Studies showed that by combining the deep CNN and temporal models, one could effectively recognize facial emotion from the videos [50,52]. FaceNet is a deep CNN model that has been utilized to identify facial features from the image inputs [53]. FaceNet has an open-source library (https://github.com/timesler/facenet-pytorch, accessed on 18 July 2021) implemented with Pytorch designed for face verification, recognition, and feature extraction. The Multitask CNN (MTCNN) [54] recognizes a face in the video frames and returns the sequence of the cropped images with the detected face from the video input. Afterward, we used the Inception ResNet (V1) model, which was pre-trained on the VGGFace2 [55] and CASIA-Webface [56] datasets, to extract the sequence of face embeddings from the sequence of images. The dimension of the returned embeddings, by default, equals 512. As illustrated in Figure 2, we can obtain a sequence of face embeddings during the time interval of one person’s speech.

Furthermore, we incorporated an RNN-based model to learn the temporal relations in the sequence of images. The Gated Recurrent Unit (GRU) [57] is similar to the LSTM [58] network with a forget gate, but is more efficient in training. The formulations of the GRU network can be stated as follows:

- Update gate $z_{t}$: defines how much of the previous memory to keep.
  (2) $$z_{t}=\sigma \left(W^{z}x_{t}+U^{z}h_{t-1}\right);$$
- Reset gate $r_{t}$: determines how to combine the new input with the previous memory.
  (3) $$r_{t}=\sigma \left(W^{r}x_{t}+U^{r}h_{t-1}\right);$$
- Cell value ${\tilde{h}}_{t}$:
  (4) $${\tilde{h}}_{t}=\tanh\left(W^{h}x_{t}+U^{h}\left(h_{t-1}⊙r_{t}\right)\right);$$
- Hidden value $h_{t}$:
  (5) $$h_{t}=\left(1-z_{t}\right)⊙{\tilde{h}}_{t}+z_{t}⊙h_{t-1};$$

where ⊙ denotes the Hadamard product.

The extracted sequence of face images has variable lengths; therefore, some samples were downsampled to the fixed length. Due to the limitation of the GPU’s memory and the consideration of training efficiency, the fixed length for the extracted images sequence in this study was set to 50 images. Oppositely, zero-padding was applied during data preprocessing to the sequences with a shorter length. Finally, during training, the last hidden state value, $h_{t}$, of the GRU outputs was extracted, and a classifier was introduced to learn the emotion classification task.

### 4.2. Robust Crossmodality Fusion Transformer with EmbraceNet

**Figure 4:**
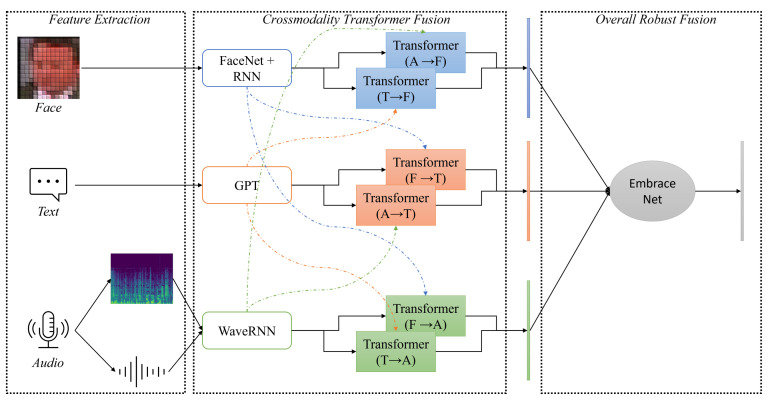
The crossmodal fusion transformer [40] with EmbraceNet [34].

In this section, we demonstrate the network architecture for multimodal fusion and classification, which is depicted in Figure 4. Our model consisted of two main parts, *crossmodality transformer fusion* and *overall robust fusion*.

#### 4.2.1. Crossmodality Transformer Fusion

The idea of the crossmodal transformer was initially proposed by Tsai et al. [40]. The crossmodal transformer can enrich the information for one modality from another modality. In this study, we adapted the network architecture to fuse different input modalities for emotion recognition. Following the formulation of [40], for example, we denote the passing of Modality A information to another Modality B by using “A→B”. The multi-head attention was proposed in the work [35], where the attention function was mapping the query, keys, and values to the output.

**Figure 5:**
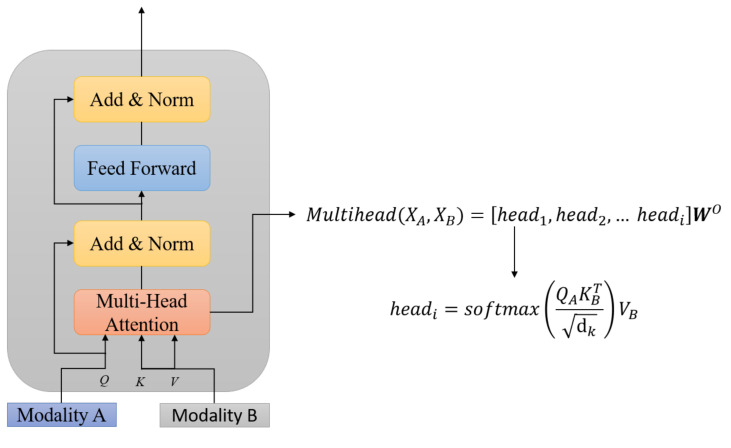
The architecture of the crossmodal transformer.

Figure 5 shows the architecture of the crossmodal transformer. We denote the input features for Modalities A and B as $X_{A}\in R^{T_{A}\times d_{A}},X_{B}\in R^{T_{A}\times d_{B}}$, where *T* and *d* are the sequence length and feature size. As shown in Figure 5, we can define the query as $Q_{A}=X_{A}W_{Q_{A}}$, keys as $K_{B}=X_{B}W_{K_{B}}$, and values as $V_{B}=X_{B}W_{V_{B}}$, where $W_{Q_{A}}\in R^{d_{A}\times d_{k}},W_{K_{B}}\in R^{d_{B}\times d_{k}},W_{V_{B}}\in R^{d_{B}\times d_{V}}$ are the weights. Then, the fused attention output vector *Y* from Modality *A* to *B* can be represented as follows:

(6) $$\begin{array}{cc} Y_{attention} & =Attention(X_{A},X_{B})\\ \; & =softmax\left(\frac{X_{A}K_{B}^{⊺}}{\sqrt{d_{k}}}\right)V_{B}\\ \; & =softmax\left(\frac{X_{A}W_{Q_{A}}W_{K_{B}}^{⊺}X_{B}^{⊺}}{\sqrt{d_{k}}}\right)X_{B}W_{V_{B}}. \end{array}$$

Following the setting from the previous study [35], we also added a residual connection from the query to the attention output and layer normalization.

(7) $$x=LayerNorm(Y_{attention}+Q_{A}).$$

Then, a feed-forward layer was applied, which consisted of two fully connected layers with a ReLU activation function:

(8) $$f_{x}=Linear\left(x\right)=xA_{x}^{⊺}+b_{x}.$$

(9) $$x_{a}=ReLU\left(f_{x}\right).$$

(10) $$f_{x_{a}}=Linear\left(x_{a}\right)=x_{a}A_{x_{a}}^{\prime }{}^{⊺}+b{}_{x}{}_{a},$$

where $A_{x}\in R^{d_{x}\times 2d_{x}}$, $A_{x_{a}}^{\prime }\in R^{2d_{x}\times d_{x}}$, and $b_{x}\in R^{2d_{x}}$, $b_{x}\in R^{d_{x}}$.

Finally, another residual connection with normalization was used to obtain the final embedding representation vector from Modality *A* to *B*.

(11) $$out_{A\to B}=LayerNorm\left(f{}_{x_{a}}+x\right).$$

Taking the example of the crossmodal fusion for the text modality, the final attention representation of the feature was the Hadamard product of two crossmodality features, as suggested in previous study [38], which is given as follows:

(12) $$Attention_{T}=out_{F\to T}⊙out_{A\to T}.$$

where A denotes audio, F denotes face, T denotes text, and ⊙ denotes the Hadamard product.

During training, the crossmodal transformer module transforms the source modality into the key/value pair to interact with the query, the target modality. The previous study showed that the crossmodal transformer can learn correlated information across modalities [40].

#### 4.2.2. EmbraceNet for Robust Multimodal Fusion

For the multimodal emotion recognition task, we not only considered crossmodal fusion by using the transformer but also wanted to ensure the robustness of combining the multimodal outputs. We employed the EmbraceNet [34] in our network architecture, which focuses on dealing with crossmodal information carefully and avoids performance degradation due to the partial absence of data.

**Figure 6:**
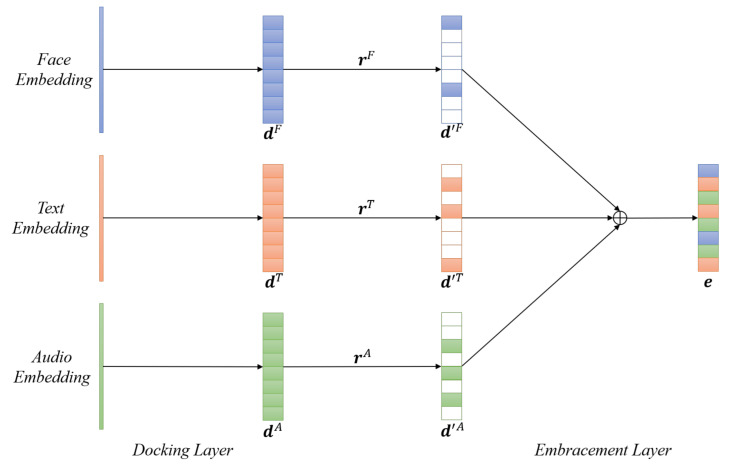
The EmbraceNet for multimodal fusion.

As can be seen in Figure 6, the EmbraceNet consists of two main components, the docking layers, and an embracement layer.

#### 4.2.3. Docking Layers

Three different deep learning models were used to obtain the feature vectors from the three different modalities. Each modality has different characteristics and different sizes of extracted feature vectors, so the docking layers act as a preprocessing module before being fed into the embracement layer by converting different modalities’ features to the same size. The docking layers consist of a fully connected layer followed by a ReLU activation function. Each feature vector from different modalities is converted to the same embracement size, which in this study was equal to 256. Finally, the docking layers output *m* feature vectors, $d^{k}\in \left\{d^{\left(1\right)},d^{\left(2\right)},\ldots ,d^{\left(m\right)}\right\}$, where m=3 denotes the number of modalities in this study, and $d^{k}={\left[d_{1}^{\left(k\right)},d_{2}^{\left(k\right)},\ldots ,d_{c}^{\left(k\right)}\right]}^{⊺}$, where *c* is the dimensionality of the vector.

#### 4.2.4. Embracement Layer

The embracement layer is formalized as follows. Let $r_{i}=\left[r_{i}^{\left(1\right)},r_{i}^{\left(2\right)},\ldots r_{i}^{\left(m\right)}\right]$, where i∈{1,2,…,c} and m∈{1,2,3}, be a vector that can be drawn from a multinomial distribution, i.e.,

(13) $$r_{i}∼\mathrm{Multinomial}\left(1,p\right),$$

where $p={\left[p_{1},p_{2},\ldots ,p_{m}\right]}^{⊺}$ are the probability values and $\sum_{k}p_{k}=1$. It should be noted that only one value of $r_{i}$ is equal to 1, and the rest of the values are 0. Then, the vector $r_{i}$ is applied to $d^{k}$ as:

(14) $$d^{\prime }\left(k\right)=r^{\left(k\right)}⊙d^{\left(k\right)},$$

where ⊙ denotes the Hadamard product (i.e., $d^{\prime }{}_{i}{}^{\left(k\right)}=r{}_{i}{}^{\left(k\right)}\cdot d{}_{i}{}^{\left(k\right)}$). Finally, the model combines all the features to generate a fused embedding vector $e={\left[e_{1},e_{2},\ldots ,e_{c}\right]}^{⊺}$, and:

(15) $$e_{i}=\sum_{k}d^{\prime }{}_{i}^{\left(k\right)},$$

where e is the final output vector for the final multimodal fusion emotion classification task. In this study, k∈{F,T,A}, and the final representation of the output vector is:

(16) $$e_{i}=\sum_{k\in \{F,T,A\}}d^{\prime }{}_{i}^{\left(k\right)}.$$

As stated in [34], the docking layers that consider the correlations between different modalities during training cause only part of the feature embedding vector for each modality to be further processed in the embracement layer. The selected features’ indices are randomly changed so that each docking layer will learn to generate similar embedding vectors, and the embracement layer can generate the same output. The multinomial distribution for the selection process also acts as a regularization step, preventing the model from excessively learning from specific modalities.

## 5. Experiments

### 5.1. Computational Environment

Pytorch (Version 1.8) with CUDA Version 10.2 was utilized to develop the model and evaluate the performance of the MELD. The training of the model was run on two Nvidia GeForce GTX 2080 Ti graphic cards with 11 GB of memory.

### 5.2. Training Details

The training process consisted of two parts. As stated above, we first fine-tuned the single modality data via three different domain-specific models. For the text modality, the inputs were the word embeddings of the sentence, including the history of dialogue and the reply. Secondly, the input for the video modality was a sequence of face images with a fixed sequence length. Finally, the input for the audio modality was a combination of an audio waveform and spectrogram data for the audio modality.

Furthermore, for the proposed multimodal fusion model, we combined all of the extracted features from the domain-specific models before the last fully connected layers. We used the Stochastic Gradient Descent (SGD) optimizer with a learning rate of 0.001 and the cross-entropy loss for the multiclass classification problem.

### 5.3. Evaluation Metrics

We evaluated the performance of multimodal emotion recognition task using the following evaluation metrics: *Accuracy*, *Balanced Accuracy*, *Precision*, *Recall*, and *F1*-score. Using the notions of True Positive (TP), True Negative (TN), False Positive (FP), and False Negative (FN), the expression of these metrics is given as follows:

(17) $$Accuracy=\frac{TP+TN}{TP+FP+TN+FN},$$

(18) $$Balanced\;Accuracy=\frac{Sensitivity+Specificity}{2},$$

(19) $$Precision=\frac{TP}{TP+FP},$$

(20) $$Recall=\frac{TP}{TP+FN},$$

(21) $$F1=2\frac{Precision\times Recall}{Precision+Recall},$$

where:

(22) $$Sensitivity=\frac{TP}{TP+FN},$$

(23) $$Specificity=\frac{TN}{TN+FP}.$$

It should be noted that the *Balanced Accuracy* is commonly used for evaluating imbalanced datasets; thus, it was believed that it would be effective for evaluating the MELD. *Balanced Accuracy* for multiclass classification is defined as the average *Recall* obtained from each of the classes. The *Precision* shows how many positive predicted samples are truthfully positive, and the *Recall* tells how many positive samples are correctly classified as positive by the model. The *F1*-score takes both the Precision and Recall into account, which is the harmonic mean of the *Precision* and *Recall*.

### 5.4. Performance Evaluation

We evaluated the performance of our fusion model via the following strategies: (1) comparing the performances of the classification for the unimodal and for the multimodal models and (2) contrasting the performances of our proposed method with existing studies. The visualization of the performance includes using a confusion matrix and the feature embedding visualization of the MELD using t-distributed Stochastic Neighbor Embedding (t-SNE) [59].

## 6. Results and Discussion

### 6.1. Performance Comparison between Single Modality and Multiple Modalities

Since the MELD has already been divided into training, validation, and test sets, we built our crossmodality fusion model, tuned the training hyperparameters based on the training and validation set, and evaluated the model on the test set to obtain the final results. Table 1 shows the performances of the single modality and the proposed multimodal fusion model. The fusion evaluation was performed by evaluating the classification results of the fused representation vectors. The fusion model outperformed all single modality models from the weighted average metrics. Specifically, the proposed fusion model achieved a *Precision* of 63.1%, a *Recall* of 65.0%, and an *F1*-score of 64.0% based on the weighted average. The text modality model contributed the most to the final fusion results, achieving a weighted average *F1*-score of 61.8%.

Table 2 presents the overall performance of the single modality and multimodal models. Evaluating the performance of each individual modality, the modality of text had the highest *Accuracy* and *Balanced Accuracy* from the experiments. However, the face modality had the lowest results. All evaluation metrics showed that our multimodal fusion model outperformed the unimodal results.

**Figure 7:**
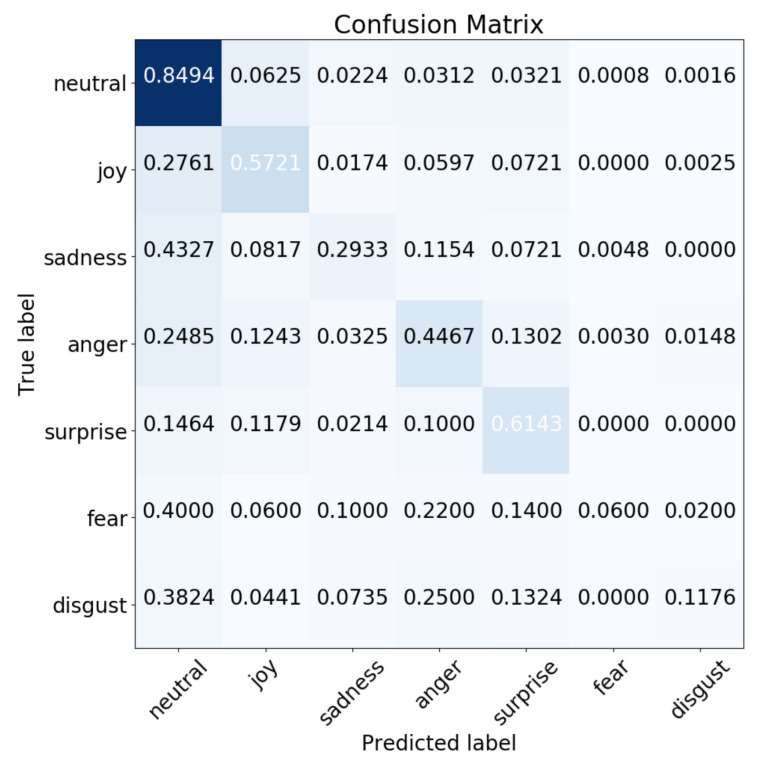
The confusion matrix for the multimodal results.

The inherent imbalance issue from the MELD caused the low performance of the *Balanced Accuracy*, which is also reflected in the confusion matrix of the multimodal results. As can be seen in Figure 7, most of the predictions of samples tend to lie in the first column of the confusion matrix, which is the *Neutral* emotional class. The reason for this issue is that over 47% of the samples from the MELD are labeled as *Neutral*. Therefore, the model is influenced by the data imbalance and learns more weights for the *Neutral* emotional class.

### 6.2. Comparison with Existing Studies

Table 3 compares the performance of the proposed model with the existing studies that also implemented the multimodal architecture and tested it on the MELD. Most of the previous studies [36,39] only considered the audio and text modalities. However, the study by Siriwardhana et al. [38] proposed a multimodal fusion model for combining the modality of audio, face, and text and achieved state-of-the-art results. The results from the previous studies demonstrated that both the *Accuracy* and *F1*-scores were improved by combining multiple modalities compared with a single modality. The previous results also yielded that single modality models for both audio and face modalities cannot learn any distinct emotion features, which is also supported by the results of our experiments. Compared to the study by [38], our proposed method achieves higher *Accuracy* and equivalent *F1*-scores, which matches the state-of-the-art performance and propounds the robustness in multimodal emotion classification.

### 6.3. t-SNE Visualization for the MELD

**Figure 8:**
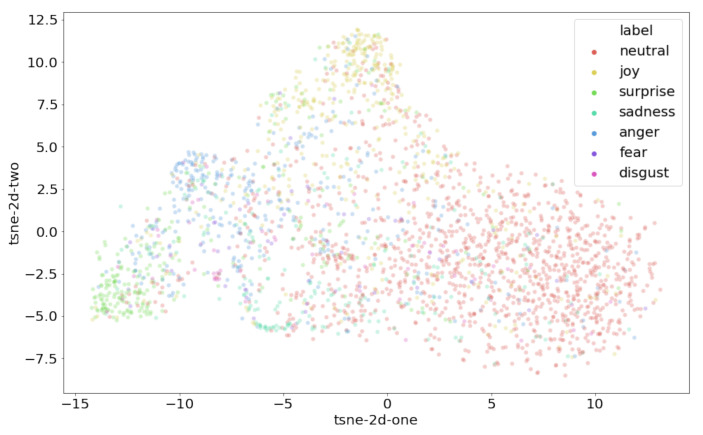
The t-SNE visualization for the MELD.

Figure 8 shows the visualization of the embedding outputs from the last fully connected layer of our proposed model. The embedding vectors are projected into a 2D plan by using t-SNE [59]. As can be seen, the sparseness of the *Neutral* class spans over all the other classes, which makes training more challenging. However, this is not particularly surprising given the fact that the *Neutral* emotion may contain characteristics from either emotion.

It can also be seen that the cluster of the *Surprise* emotion is far away from the cluster of *Neutral* emotion data points, meaning the model generates distinct features for this class, which are also reflected in Table 1, showing that the *Surprise* emotion obtains the highest *F1*-score among the other emotion classes, except for *Neutral*.

## 7. Conclusions

This study demonstrated a robust multimodal emotion classification architecture, which included crossmodal transformer fusion to combine three different modalities of the input information. The architecture considers both the joint relations among the modalities and robustly fuses different sources of the representation vector. Three separate prediction models were adapted to identify emotion from audio, visual, and textual inputs. Text, audio, and image inputs were trained by GPT, WaveRNN, and FaceNet+GRU, respectively. The designed transformer-based fusion mechanism with EmbraceNet demonstrated the ability to solve the task of multimodal feature fusion from multiple pre-trained models. EmbraceNet takes the attention outputs from the crossmodal models and embraces them to build a fused representation of the emotion embedding vectors. The experiment’s metrics showed that our multimodal classification model outperforms every single modality model. However, due to innate imbalance in the dataset, *Balanced Accuracy* is generally lower than *Accuracy*. Future studies should consider introducing data augmentation techniques to handle the imbalanced data issue.

Furthermore, experimental results on the MELD demonstrated the effectiveness of the proposed method. The performance of our method can reach the previous state-of-the-art strategies [38] (with a 0.7% performance improvement on *Accuracy*). The performance of the *F1*-score is equivalent. Nevertheless, our proposed network architecture extends the previous studies of multimodal emotion recognition with the crossmodal transformer [38,40], and the structure of the network can also be robustly expanded for a higher number of input modalities. For future studies, other input modalities such as different physiological measurements should also be added to this network architecture. Besides, the emotions stimulated by actors from a comedy can be exaggerated and different from real emotions, which could also lead to biased results [60]. Therefore, more multimodal datasets should be evaluated in future work.

## Figures and Tables

**Table 1:** The performance of the classification results for the MELD (PRE: Precision, REC: Recall, F1: F1-score) (Unit = %).

Emotion	Modality											
	Audio			Face			Text			Multimodal		
	PRE	REC	F1	PRE	REC	F1	PRE	REC	F1	PRE	REC	F1
Neutral	48.5	99.8	65.2	48.1	99.0	64.7	72.7	82.1	77.1	74.0	84.9	79.1
Joy	45.5	1.2	2.4	15.0	0.7	1.4	54.4	53.7	54.1	56.7	57.2	56.9
Sadness	0.0	0.0	0.0	0.0	0.0	0.0	40.1	33.2	36.3	49.6	29.3	36.9
Anger	60.0	0.9	1.7	0.0	0.0	0.0	53.4	39.1	45.1	51.4	44.7	47.8
Surprise	16.7	0.4	0.7	0.0	0.0	0.0	53.0	60.0	56.3	54.4	61.4	57.7
Fear	0.0	0.0	0.0	0.0	0.0	0.0	16.1	10.0	12.3	50.0	6.0	10.7
Disgust	0.0	0.0	0.0	0.0	0.0	0.0	52.4	16.2	24.7	47.1	11.8	18.8
Weighted Avg.	40.0	48.4	43.8	25.5	47.8	33.3	61.0	62.6	61.8	**63.1**	**65.0**	**64.0**

**Table 2:** The overall classification results of the single modality and multimodal methods (Unit = %).

Modality	Accuracy	Balanced Accuracy	F1
Audio	48.4	14.6	43.8
Face	47.8	14.3	33.3
Text	62.6	42.0	61.8
Multimodal	65.0	42.2	64.0

**Table 3:** The comparison of the proposed model with existing studies (A: Audio, F: Face, T: Text) (Unit = %).

Model	Modality	Acc	F1
N. Ho et al. [36]	A	48.8	45.3
	T	61.7	59.0
	Multimodal (A + T)	63.3	60.6
Z. Lian et al. [39]	Audio	46.9	38.2
	Text	60.6	58.3
	Multimodal (A + T)	62.0	60.5
S. Siriwardhana et al. [38]	Multimodal (A + F + T)	64.3	63.9
Robust Crossmodality Fusion (proposed)	Audio	48.4	32.1
	Face	47.8	31.4
	Text	62.6	61.2
	Multimodal (A + F + T)	**65.0**	**64.0**

## Data Availability

The MELD dataset used in this study can be found at https://affective-meld.github.io/ (accessed on 18 July 2021).
